# Host Response and Bacterial Virulence Factor Expression in *Pseudomonas aeruginosa* and *Streptococcus pneumoniae* Corneal Ulcers

**DOI:** 10.1371/journal.pone.0064867

**Published:** 2013-06-04

**Authors:** Rajapandian SivaGanesa Karthikeyan, Jeganathan Lakshmi Priya, Sixto M. Leal, Jonida Toska, Arne Rietsch, Venkatesh Prajna, Eric Pearlman, Prajna Lalitha

**Affiliations:** 1 Dr. G. Venkatasamy Eye Research Institute, Aravind Eye Hospital, Madurai, Tamil Nadu, India; 2 Department of Ophthalmology and Visual Sciences, Case Western Reserve University, Cleveland, Ohio, United States of America; 3 Department of Molecular Biology and Microbiology, Case Western Reserve University, Cleveland, Ohio, United States of America; Duke University Medical Center, United States of America

## Abstract

*P. aeruginosa* and *S. pneumoniae* are major bacterial causes of corneal ulcers in industrialized and in developing countries. The current study examined host innate immune responses at the site of infection, and also expression of bacterial virulence factors in clinical isolates from patients in south India. Corneal ulcer material was obtained from 49 patients with confirmed *P. aeruginosa* and 27 patients with *S. pneumoniae*, and gene expression of Toll Like Receptors (TLR), cytokines and inflammasome proteins was measured by quantitative PCR. Expression of *P. aeruginosa* type III secretion exotoxins and *S. pneumoniae* pneumolysin was detected by western blot analysis. We found that neutrophils comprised >90% cells in corneal ulcers, and that there was elevated expression of TLR2, TLR4, TLR5 and TLR9, the NLRP3 and NLRC4 inflammasomes and the ASC adaptor molecule. IL-1α IL-1β and IFN-γ expression was also elevated; however, there was no significant difference in expression of any of these genes between corneal ulcers from *P. aeruginosa* and *S. pneumoniae* infected patients. We also show that 41/49 (84%) of *P. aeruginosa* clinical isolates expressed ExoS and ExoT, whereas 5/49 (10%) of isolates expressed ExoS, ExoT and ExoU with only 2/49 isolates expressing ExoT and ExoU. In contrast, all 27 *S. pneumoniae* clinical isolates produced pneumolysin. Taken together, these findings demonstrate that ExoS/T expressing *P. aeruginosa* and pneumolysin expressing *S. pneumoniae* predominate in bacterial keratitis. While *P. aeruginosa* strains expressing both ExoU and ExoS are usually rare, these strains actually outnumbered strains expressing only ExoU in the current study. Further, as neutrophils are the predominant cell type in these corneal ulcers, they are the likely source of cytokines and of the increased TLR and inflammasome expression.

## Introduction

Microbial infections are an important cause of corneal ulcers worldwide, with *Pseudomonas aeruginosa* and *Streptococcus pneumoniae* infections resulting in severe corneal opacity, ocular pain and visual impairment [1]–[3]. Although *P. aeruginosa* is the most frequent cause of severe microbial keratitis worldwide, *S. pneumoniae* keratitis is a major cause of corneal ulcers in India and other developing countries [1], [3], [4]. Predisposing factors include contact lens wear in industrialized countries, whereas trauma to the ocular surface is the most common risk factor in developing countries [1]–[3]. In southern India, corneal injury accounts for 70% of fungal and bacterial infections, primarily as a consequence of agricultural related trauma [1], [2]. Although polymicrobial infections are occasionally detected, they comprise <2% of total cases, with the vast majority of corneal ulcers are associated with a single organism, either fungal or bacterial [2], [5].

We recently characterized the host response in patients with corneal ulcers caused by the fungal pathogens *Aspergillus* and *Fusarium*, and showed elevated expression of pro-inflammatory and chemotactic cytokines, in addition to pathogen recognition molecules including Toll Like Receptors (TLR) and c-type lectins [6]. In the current study we examined the host response in corneal ulcer material from patientes infected with *P.aeruginosa* or *S. pneumoniae.* In addition, we examined bacterial virulence factors in clinical isolates, focusing on expression of *S. pneumoniae* pneumolysin, and *P.aeruginosa* exotoxins (ExoS, ExoT, ExoU) exported by the Type III secretion system. We report that all of the *S. pneumoniae* clinical isolates express pneumolysin, and that all of the *P.aeruginosa* isolates express exotoxins, with ExoS expressing strains comprising 84%. Taken together with our data on innate immunity, these findings, add to our understanding of the pathogenesis of bacterial keratitis in infected individuals.

## Materials and Methods

### Ethics Statement

The protocol for obtaining corneal ulcer scrapings was reviewed and approved by the Internal Institutional Review Board of the Aravind Medical Research Foundation. The aims and methodology of the research were thoroughly explained to the patients, and the samples were collected after obtaining informed consent. Patients with acute or chronic systemic illness or with any form of immunosuppression or topical steroid therapy were excluded from this study. All studies on patient material were performed in India.

### Bacterial Strains

*Pseudomonas aeruginosa* strain PA01 expresses ExoS and ExoT, whereas PA103 expresses ExoT and ExoU [7]–[9]. Both strains were maintained in the laboratory of AR. The pneumolysin expressing *Streptococcus pneumoniae* ATCC-49619 reference strain was obtained from the American Type Culture Collection (Mannasas, VA).

### Identification of Bacteria in Corneal Ulcers

Corneal ulcer material was collected aseptically using a sterile kimura spatula. Scrapings were placed directly onto separate glass microbiology slides for Gram stain and further scrapings were taken and directly inoculated onto sheep’s blood agar, chocolate agar, potato dextrose agar or Sabouraud’s agar for bacterial and fungal culture. An additional sample was collected in TRIzol reagent (Invitrogen, Carlsbad, CA) to extract RNA. The inoculated plates were incubated overnight, and bacteria were identified based on colony morphology on solid media, *Streptococcus* haemolytic activity on blood agar, and was confirmed using Analytical Profile index biochemical strips (BioMerieux India Pvt. Ltd). The DNA from the pure culture of the pathogens was subjected to the 16s rDNA sequencing using the ABI Genetic analyzer 3130.

### RNA Extraction, cDNA Conversion and Quantitative PCR Analysis

Corneal scrapings from patient ulcers were homogenized in TRIzol (Invitrogen, Carlsbad, CA), using a handheld homogenizer (Labware Scientific, USA), and total RNA was extracted from corneal tissue samples according to the manufacturer's directions followed by DNase treatment (Invitrogen, Carlsbad, CA). The quality of RNA was checked by agarose gel electrophoresis, and 260/280 ratio, which was determined using a Nano drop spectrophotometer. RNA with a ratio >1.8 was converted into cDNA.

cDNA was generated using the SuperScript First Strand synthesis system (Invitrogen) using standard methods. After cDNA conversion the RNA were digested with RNaseH (Invitrogen, Carlsbad, CA) for 20 min at 37°C, and quantitative PCR was performed using the SYBR green system (Applied Biosystems, Carlsbad, CA). Primer sequences were designed by using the NCBI Primer BLAST or downloaded from Primer bank (**Table 1**), and synthesized at Bioserve India Ltd, Hyderabad, India. Universal PCR conditions were utilized for cDNA amplification for all primer sets. Melting curve analysis was performed to confirm specific gene amplification, and PCR product sizes were also confirmed by agarose gel electrophoresis.

**Table 1:** Primer sequences.

Gene	Gene NCBI geneid/Primer Bank id	Forward Primer Sequence (5′->3′)	Reverse Primer Sequence (3′->5′)	Product size
TLR2	NM_003264.3	CTTCACTCAGGAGCAGCAAGCA	ACACCAGTGCTGTCCTGTGACA	146
TLR4	NM_138554.3	CCCTGAGGCATTTAGGCAGCTA	AGGTAGAGAGGTGGCTTAGGCT	126
TLR5	16751843a2	CAGAAACCTGCCCAACCTTAG	GATCCAAGCGAGTTAAAGCCTT	182
TLR9	9887085a2	GGAAGAGCTAAACCTGAGCTACA	GGGATATGAGGGATTTGGGCA	68
NLRP3	20268804a3	TAGCCACGCTAATGATCGACT	TTGATCGCAGCGAAGATCCAC	76
ASC	10835256a3	TGACGGATGAGCAGTACCAG	GCTTCCGCATCTTGCTTGG	63
IL-1α	27894330a2	GAAGAGACGGTTGAGTTTAAGCC	CAGGAAGCTAAAAGGTGCTGA	112
IL-1β	NM_000576.2	CCACAGACCTTCCAGGAGAATG	GTGCAGTTCAGTGATCGTACAGG	131
IFN-γ	NM_000619.2	GAGTGTGGAGACCATCAAGGAAG	TGCTTTGCGTTGGACATTCAAGTC	124
β-actin		CCTGGCACCCAGCACAAT	GGGCCGGACTCGTCATAC	126

The quantification cycle of the target gene was normalized using β-actin, and the fold change with respect to non-infected, donor corneas was calculated using the 2^−ΔΔct^ method. Data are presented as log of relative gene expression (log(RQ)) as described in our previous study [6].

### Characterization of Host Cells in Corneal Tissues

Infected corneal ulcer material was spread on a standard microscope slide and stained with the modified Wrights Giemsa solutions (Diff-Quik) to identify neutrophils and mononuclear cells. Perentage was determined from ten *P. aeruginosa* and ten *S. pneumoniae* infected patients. The slides were also Gram stained to detect bacteria.

### Expression of Streptolysin and *P. aeruginosa* Exoenzymes

*P. aeruginosa* clinical isolates were grown in Luria-Bertani (LB) broth with 200 mM NaCl, 10 mM MgCl_2_, and 0.5 mM CaCl_2_. 5 mM EGTA (Sigma) was added to chelate calcioum and induce Type three secretion,. *S. pneumonia* strains were grown in 250 ml of brain heart infusion broth (Hi-Media, Bombay, India) supplemented with 0.5% yeast extract, casein and 5% CO_2_ at 37°C for 20 hours. Cells were harvested by centrifugation at 6400×g for 10 minutes. The clinical isolates and the lab strains PAO1 and PA103 were grown to an OD_600_ of 0.6 in the presence of calcium, centrifuged and resuspended 30 min in medium without calcium. The cell supernatant was precipitated with 10% trichloroacetic acid (TCA) followed by acetone wash. The cell pellet and TCA-precipitated supernatant protein were separated by 12% SDS-PAGE gel, and blotted to nitrocellulose membrane, and probed with the corresponding rabbit antibody for each exoenzyme. The anti-ExoU antibody was a kind gift from Dr. Alan Hauser (Northwestern Univeristy). Anti-Pneumolysin antibody sc-80500 was purchased from Santa Cruz Biotechnology (Dallas, TX). HRP-conjugates secondary antibodies were detected using a chemiluminense kit per manufacturer’s instructions (GE Health care, Amersham, Piscataway, (NJ).

### Statistical Analysis

Differences in gene expression between normal, uninfected donor corneas and infected corneas, and between *Pseudomonas aeruginosa* and *Streptococcus pneumoniae* infected corneal tissue were calculated by Analysis of Variance with a post-hoc Tukey test, or using an unpaired t test using GraphPad Prism software (LaJolla, CA), and statistical significance was defined as p<0.05.

## Results

### Study Population

The Aravind Eye Hospital is a primary eye care facility in Madurai, Tamil Nadu, which is routinely involved in the diagnosis and treatment of patients with corneal ulcers. Patients in the current study reported to the hospital within one week after a traumatic event to the cornea, which was most often associated with corneal injury from plant or soil material, and presented with severe pain, conjunctival vascularization, and photophobia (**Table 2**). Patients who participated in the study had no systemic illness or immunosuppressive therapy.

**Table 2:** Clinical characteristics.

Characteristic	*P. aeruginosa*	*S. pneumoniae*
**Age, Years**		
Range	7 to 70	14 to 72
Mean (SD)	44.9±17.4	46.1±16.4
**Sex**		
Male	10 (47.62)	16 (59.26)
Female	11 (52.38)	11 (40.74)
Total	21 (100.00)	27 (100.00)
**Days since initial trauma**	5.5±4.1	10.9±14.0
**Size of the ulcer mm^2^**		
<5 (number, percent total patients)	3 (14.29)	9 (33.33)
5 to 10	8 (38.10)	9 (33.33)
10 to 14	8 (38.10)	4 (14.81)
>14	2 (9.52)	5 (18.52)
**Location of the ulcer**		
Central	7 (33.33)	9 (33.33)
Paracentral	12 (57.14)	13 (48.15)
Total	2 (9.52)	5 (18.52)
**Depth of the ulcer**		
Superficial	3 (14.29)	4 (14.81)
Mild	8 (38.10)	9 (33.33)
Deep	10 (47.62)	14 (51.85)
**Hypopyon** *		
Yes	15 (71.43)	23 (85.19)
No	6 (28.57)	4 (14.81)
**Clinical Outcome**		
Healed	15 (71.43)	22 (81.48)
Treatment failure	2 (9.52)	5 (18.52)
No follow-up	4 (19.05)	NA
**Visual acuity**		
Improved	8 (38.10)	17 (62.96)
No change	4 (19.05)	3 (11.11)
No follow-up	4 (19.05)	1 (3.70)
Worse	5 (23.81)	6 (22.22)

Data are number and percent (%) of patients, unless otherwise indicated. Patients (n = 48) had corneal ulcers and presented at the clinic within 1–2 weeks after infection; corneal scrapings from the ulcer were used in the present study. Ten donor corneas from individuals with no infection or inflammation were obtained from the International Rotary Aravind Eye Bank. NA, not applicable.

* accumulation of neutrophils in the anterior chamber.

As shown in Table 2, *P. aeruginosa* was identified in 21 patients, and *S. pneumonia* was identified in 27 corneal ulcers. Patients were males and females between 7 and 72 years old. The size and depth of ulcers among the patients ranged from <5 mm^2^ to >14 mm^2^ in area. The majority (>70%) of bacterial ulcers eventually healed following antibiotic treatment; however a few patients with large ulcers >10 mm^2^ with deep stromal infection failed to respond to the antibiotic treatment and underwent corneal transplantation (**Table 2**). As control corneal tissue, ten non-infected donor corneas were obtained from the International Rotary Aravind Eye bank. Donors were aged 62.8±7.2 (mean +/− SD) who had died of natural causes and had no history of corneal infection or other ocular disease.

### Clinical Appearance and Cellular Infiltration in *P. aeruginosa* and *S. pneumoniae* Keratitis

**Figure 1:**
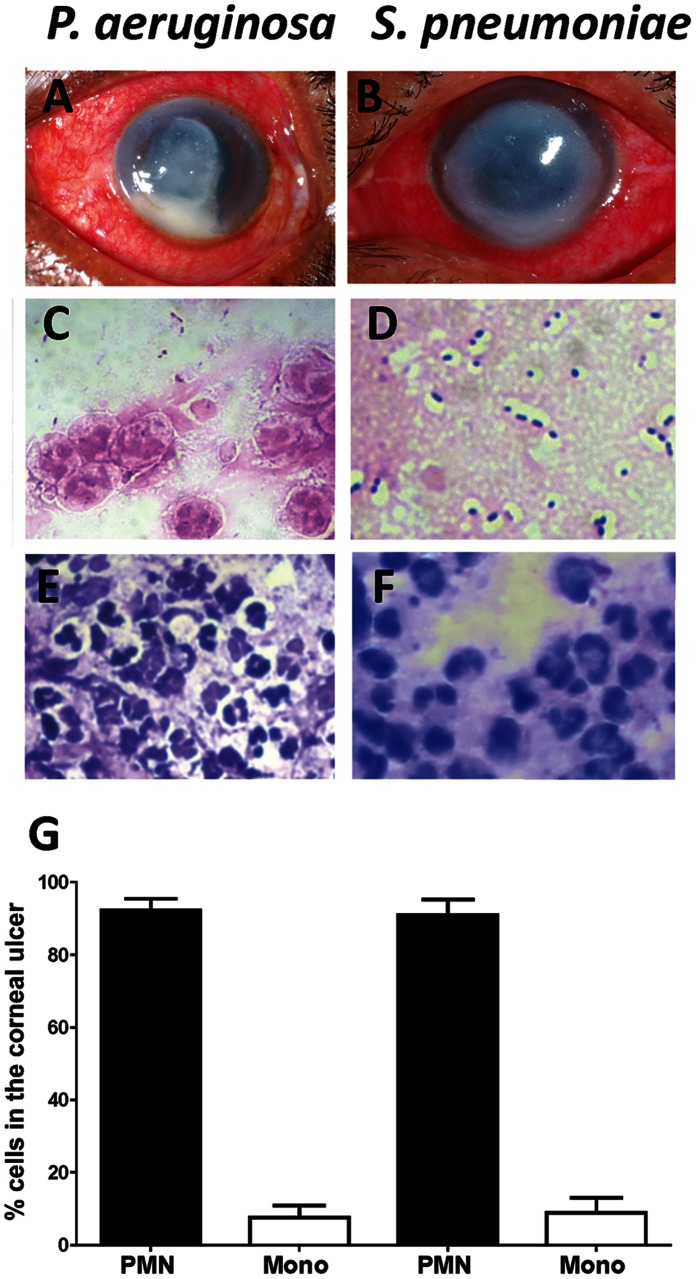
Cellular composition of corneal ulcers from patients with bacterial keratitis. Representative corneal ulcers of patients caused by *P. aeruginosa* (A), or by *S. pneumoniae* (B). C, D. Gram staining of corneal ulcer material showing Gram negative bacilli (C), and Gram positive diplococci and chains (D). Original magnification is x1000. E,F: Wrights Giemsa (Diff-Quik) stain of corneal ulcer material from *P. aeruginosa* (E), or *S. pneumoniae* (F) infected tissue Original magnification is x400. G. Percent neutrophils and mononuclear cells were determined by counting cells from ten *P. aeruginosa* and ten *S. pneumoniae* patients.

**Figures 1A, B** are representative corneas of patients with culture proven *P. aeruginosa* and *S. pneumoniae* keratitis, showing severe corneal opacification and ulceration, and conjunctival inflammation. Examples of Gram stained corneal ulcer material show Gram negative rods typical of *P. aeruginosa* (**Figure 1C**), and Gram positive diplococci and chains (**Figure 1D**), which are indicative of *S. pneumoniae.* **Figures 1E,F** show polymorphonuclear cells that are characteristic of neutrophils, although mononuclear and epithelial cells were also detected. Neutrophils were found to comprise 92% infiltrating cells, with 5% being mononuclear cells (**Figure 1G**).

### Innate Immunity in *P. aeruginosa* and *S. pneumoniae* Keratitis

**Figure 2:**
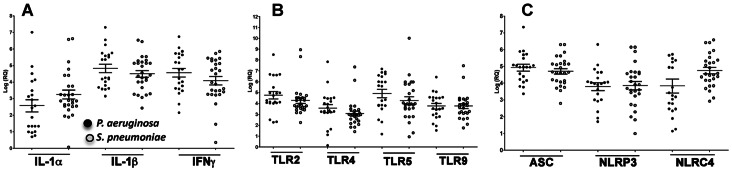
Gene expression of Toll Like Receptors, inflammasome proteins and cytokines in corneal ulcers from patients with bacterial keratitis. RNA was extracted from corneal ulcers, reverse transcribed and processed for Q-PCR. Data points represent individual patients infected with *P. aeruginosa* (closed circles) or *S. pneumoniae* (open circles), and the values presented are the log of relative gene expression (log(RQ)) in relation to uninfected donor corneas calculated using the 2^−ΔΔct^ method described in Methods. A. Pro-inflammatory cytokines IL-1α, IL-1β and IFN-γ; B. Toll Like Receptors and C. Inflammasome proteins. There were no significant differences in gene expression between *P. aeruginosa* and *S. pneumoniae* (p>0.05).

Toll like receptors (TLR) and IL-1β are expressed in corneal ulcers caused by filamentous fungi [6], and mediate disease pathogenesis and bacterial killing in murine models of *Pseudomonas* keratitis [10], [11]. To examine expression of innate immune genes in *P. aeruginosa* and *S. pneumoniae* corneal ulcers, RNA was extracted from 48 corneal ulcer scrapings within 1 week of trauma, reverse transcribed, and quantitative PCR was then performed. Δct data were calculated relative to β-actin, and the fold change with respect to the mean of 10 non-infected donor corneas was derived using the 2^−ΔΔCt^ method as described previously [6]. **Figure 2A** shows elevated expression of IL-1α, IL-1β and IFN-γ compared with uninfected corneas, with IL-1β and IFN-γ greater than 10,000-fold increased; however, there were no significant differences between *P. aeruginosa* and *S. pneumoniae* infected corneas, indicating that both genera of pathogenic bacteria stimulate production of proinflammatory cytokines. Similarly, expression of Toll-like Receptor 2, 4, 5 and 9 was elevated >1000-fold in *P. aeruginosa* and *S. pneumoniae* infected corneas compared with donor corneas (**Figure 2B** **)**.

As inflammasomes mediate caspase-1 dependent proteolytic cleavage of IL-1β from the 31 kD pro-form to the 17 kD mature form, we also examined expression of NOD-like receptor CARD domain-containing protein 4 (NLRC4) that is known to be activated by *P. aeruginosa* flagellin [12], [13], and NOD-like receptor protein 3 (NLRP3) and the common adaptor molecule apoptosis speck protein with caspase recruitment (ASC), which are associated with *S. pneumoniae* pneumolysin [12]–[14]. **Figure 2C** shows >1,000 fold increased expression of NLRC4, NLRP3 and ASC in all infected corneal ulcers compared with normal controls, although there were no significant differences between *P. aeruginosa* and *S. pneumoniae* infections. All the donor corneas had high ΔCt values (data not shown), indicating that endogenous expression of the proteins was minimal in uninfected corneas.

### Expression of S. pneumoniae Pneumolysin and P. aeruginosa Type III Secretion Exoenzymes in Clinical Isolates

**Figure 3:**
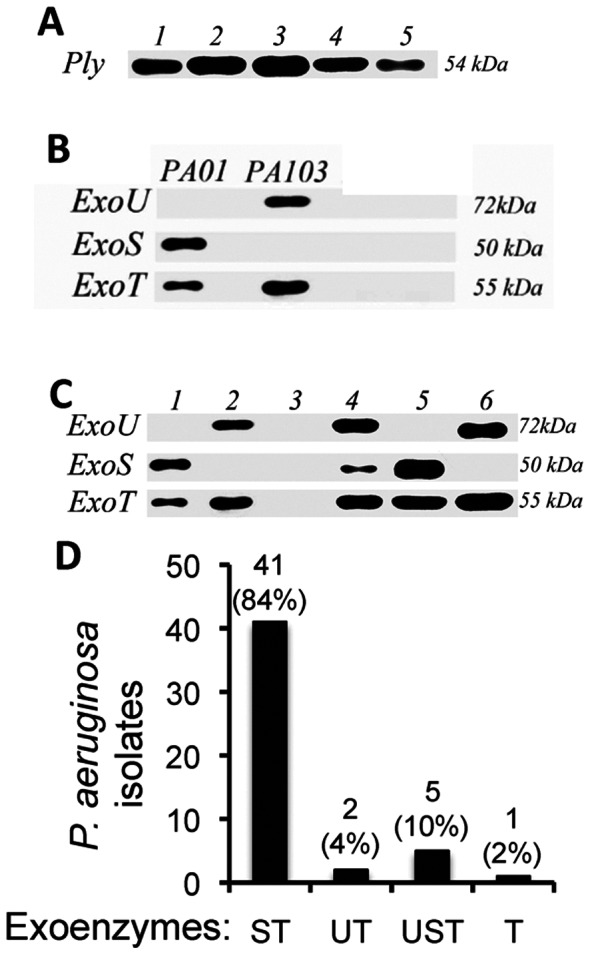
Protein expression of *S. pneumoniae* pneumolysin and *P. aeruginosa* exotoxins. A. Western blot of pneumolysin in the ATCC reference strain (Lane 1), and four representative clinical isolates (lanes 2–5). B. Western blot of culture supernatants from *Pseudomonas aerugenosa* reference strains PAO1, which expresses ExoS and ExoT, and PA103, which expresses ExoU and ExoT. C. ExoS, ExoT and ExoU expression in representative *Pseudomonas* clinical isolates. Lane 1 is similar to PAO1 in expressing ExoS and ExoT; Lane 2 is similar to PA103 in expressing ExoU and ExoT; Lane 3 is *P. otitidis*, which does not express Type III exotoxins; Lane-4 Clinical isolate express all three effector molecules; Lane-5 Exo U and Exo T expressing clinical isolate similar to PA103. D. percent and total exotoxin production by 49 clinical isolates.

Pneumolysin is a major virulence factor of *S. pneumoniae,* and pneumolysin expressing strains have been isolated from keratitis patients and shown mediate corneal disease in animal models [15], [16]. To determine if *S. pneumoniae* clinical isolates from patients at the Aravind Eye Hospital express pneumolysin, we examined bacterial lysates by Western blot analysis. As shown in **Figure 3A** **,** pneumolysin was expressed by the ATCC reference *S. pneumoniae* strain ATCC-49619 in addition to four representative clinical isolates. However, all 27 *S. pneumoniae* ocular isolates were found to express pneumolysin.

The ExoS, ExoT, and ExoU exotoxins produced by the *P. aeruginosa* Type III Secretion System play an essential role in murine models of corneal infection, partly by inducing neutrophil death and thereby inhibiting bacterial killing [17]. Further, although ExoT is co-expressed with ExoS or ExoU in environmental and clinical isolates, very few reports have identified ExoS and ExoU co-expression [18], [19].

To characterize the virulence factors expressed in the *P. aeruginosa* clinical isolates, we examined the 21 patients in Table 1 in addition to 30 archival clinical isolates. *Pseudomonas* cultures were grown under depleted calcium conditions as described in the methods, and exotoxins secreted in the culture supernatants were analyzed by Western blot. Exotoxin expression by well-characterized *P. aeruginosa* laboratory strains is shown in **Figure 3B** **,** including ExoS and ExoT production by PAO1 produced, and ExoT and ExoU expression by strain PA103. **Figure 3C** shows examples of western blots of *Pseudomonas* clinical isolates expressing ExoS, ExoT and/or ExoU, except for lane 3, which was subsequently identified as *P. otitidis*.

As shown in **Figure 3D**, among the 51 *Pseudomonas* isolates, two did not express type III effectors and were identified as *P. otitidis*. Of the 49 *P. aeruginosa* isolates, 41(84%) expressed both ExoS and ExoT, but not ExoU, two (4%) expressed ExoU and ExoT, five (10%) expressed ExoS, ExoT and ExoU and one strain expressed only ExoT. Notably, the strain expressing only ExoT also encoded *exoU* on its chromosome; however virulence in epithelial cell and animal model studies was based on expression of ExoT (data not shown), suggesting that the copy of *exoU* is not expressed or defective in this isolate.

Of the 21 *P. aeruginosa* keratitis patients, we did not find any clinical correlation with the outcome of the disease and expression of the effector molecules.

## Discussion

Although contact lens wear is associated with increased risk of developing *P. aeruginosa* keratitis [20], in India and most of the deveoping world, ocular trauma associated with agricultural work is the major risk factor underlying *P. aeruginosa* infection [1], [21]. Corneal abrasions facilitate bacterial adherence to the epithelium, and penetration to the corneal stroma. Whereas *P. aeruginosa* is ubiquitous in the environment, *S. pneumoniae* corneal infection is associated with colonization of the conjunctival sac and around the lacrimal gland [21].

We examined the host response in corneal ulcers from patients with culture positive *P. aeruginosa* or *S. pneumoniae*, and found elevated expression of the pathogen recognition receptors TLR2, TLR4 and TLR9, pro-inflammatory cytokines IL-1α, IL-1β, and IFN-γ, and the inflammasome components NLRP3, NLRC4 and ASC compared with donor corneas. As neutrophils were the predominant cell types in these corneal ulcers, they may be the source of most of these transcripts, although mononuclear cells other than corneal epithelial cells likely also contribute. Human neutrophils express all the known TLRs except TLR3, and can produce IL-1α, IL-1β, and IFN-γ, [22]. However, IL-1β secretion requires proteolytic cleavage from the inactive pro-form to the mature, secreted form. In macrophages, processing is mediated by the multi-component inflammasomes such as NLRP3/ASC and NLRC4, which activate caspase-1 mediated cleavage of IL-1β [23]. However, we showed that in a murine model of *P. aeruginosa* keratitis, neutrophils mediate IL-1β secretion using serine proteases rather than caspase-1 [10], although NLRP3 and ASC are reportedly expressed by neutrophils [24].

We found no significant differences in expression of innate immune related genes between patients infected with *P. aeruginosa* and *S. pneumoniae,* suggesting that even with the profound differences between these two bacteria, there are common factors leading to recruitment and activation of neutrophils in the cornea. Murine models of bacterial keratitis and inflammation induced by bacterial products indicate that activation of TLR ligands on resident corneal epithelial cells and macrophages induce production of chemotactic and pro-inflammatory cytokines that mediate neutrophil recruitment to the corneal stroma [11], [25]. LPS and lipoproteins will also activate TLR2 and TLR4 on neutrophils, leading to NF-κB activation and production of the same cytokines [25]. We also reported similar expression of host response genes in corneal ulcers of patients infected with either *Aspergillus* or *Fusarium* [6], indicating that there are common pathways that lead to neutrophil infiltration in fungal keratitis, most likely through activation of c-type lectins such as Dectin-1, which recognizes β-glucan on both pathogens [6], [26].

In addition to examining the host response at the site of infection, we also investigated expression of virulence factors in *P. aeruginosa* and *S. pneumoniae* isolated from corneal ulcers. We show that pneumolysin is expressed by all *S. pneumoniae* clinical isolates in the current study, indicating an essential role for this virulence factor in keratitis. Pneumolysin is likely to contribute to disease pathology both directly, by its pore-forming, cytotoxic effect on mammalian cells, which includes inserting up to 44 subunits into the cell membrane, and by activating an inflammatory response. This includes activation of NLRP3 and increased IL-1β secretion [14], [27], and although pneumolysin is a reported TLR4 ligand [28], more recent studies show that NLRP3 activation is TLR4 independent [14], [29]. Pneumolysin is released as a monomer, but intercalates into the membrane and assembles into large multimeric rings, which form pores in the cholesterol containing host cell membrane [30]. Pneumolysin also increases neutrophil degranulation and release of elastase and matrix metalloproteinases, which contribute to tissue damage in infected lungs [31], [32], and it is likely that a similar mechanism occurs in neutrophils in *S. pneumoniae* infected corneas. Also, in animal models of corneal infection, pneumolysin deficient mutants caused less severe keratitis than the parent strain, and exhibit less cytotoxicity to corneal epithelial cells [15], [16].

In the current study we also examined expression of the three main effector molecules of *P. aeruginosa*, ExoS, ExoT and ExoU, in clincial isolates. The type III secretion system of *P. aeruginosa* encodes a needle structure that injects these exotoxins directly into the host cells following contact [33]. ExoS and ExoT are highly homologous, bifunctional enzymes that contain amino-terminal Rho-GTPase-activating protein (GAP) domains with similar target specificities and carboxy- terminal ADP-ribosylation (ADPR) domains, whereas ExoU is a potent phospholipase that causes tissue destruction and inflammation [33]. A fourth exotoxin, ExoY is an adenlyate-cyclase, which does not contribute significantly to disease in animal models of infection [33]. The ADPR domain of ExoS and ExoT mediate bacterial survival and severity of disease in a murine model of *P. aeruginosa* based on strain PA01 [17], whereas phopholipase activity is essential in ExoU expressing strains [34].

In human disease, there is a correlation of the presence of specific effectors with specific diseases, in particular, ExoS-producing strains are more common in cystic fibrosis isolates, whereas ExoU-producing strains appear to be more common in isolates from keratitis patients [18], [19]. In the present study, we find that the majority of strains (84%) are ExoS+ and ExoT+, but lack ExoU. The reason for this discrepancy is unclear, but may reflect differences in the route of infection (prolonged contact lens wear compared to traumatic eye injury). Interestingly, while prior analyses indicated that strains expressing both ExoS and ExoU are a rarity, five of the seven strains expressing ExoU in our study also expressed ExoS. Although expression of effector molecules does not have any significant impact of the severity of disease in these patients, ExoS expressing vs. ExoU expressing strains have a very distinct phenotype in epithelial cells, with ExoU expressing strains having a cytotoxic phenotype causing rapid lysis, whereas ExoS expressing strains have an invasive phenotype, causing membrane blebbing within the epithelial cells as a site of bacterial replication [19], [35]. These differences extend to murine models of *P. aeruginosa* keratitis, with ExoU expressing strains causing more severe clinical disease [36]. However, in ExoS expressing strains, it is the ADPR rather than the GAP activity of the exotoxin that confers the phenotype *in vitro* and *in vivo* [17], [37]. Also, in an artificially constructed strain of *P. aeruginosa* expressing all four cytotoxins, the effect of co-expression of ExoS, ExoT and ExoY with ExoU was dependent on the inocculum: at higher infectious doses, co-expression of ExoS, ExoT and ExoY resulted in greater survival of the infected mice compared to a strain expressing ExoU alone, whereas at a lower infectious dose, the strain expressing all four effectors replicated better than the strain expressing only ExoU [38].

A recent study using a separate patient pool from a corticosteroid clinical trial found a similar distribution, with most (56/101) *P. aeruginosa* keratitis isolates expressing *exoST* genes compared with 18 expressing *exoUT*, and 27 expressing both *exoU* and *exoS* [39]. The differences in distribution likely relate to examining gene expression compared with our findings were we examined secreted exotoxins. However, results of that study also showed that patients infected with the *exoS* expressing invasive strains had a better outcome in terms of visual acuity following treatment compared with *exoU* expressing cytotoxic strains, possibly because of less severe disease [39]. Although we also found differences in exotoxin expression in clinical isolates in the current study, we did not detect clinical differences in patients, possibly because there were masked by differences in the inoculum, the time of sampling, or antibiotic treatment.

In conclusion, the current study provides direct characterization of the host response to pathogenic bacteria in infected human tissues as well as the virulence factors of these pathogens. These findings will allow us to examine the role of specific host response genes in infection and will help correlate results obtained using animal models of infection to human disease. These studies may identify potential targets for immune intervention that could regulate the severity of the host response and its effect on blindness and visual impairment caused by these organisms.
